# Microangiopathy associated with poor outcome of immunoglobulin A nephropathy: a cohort study and meta-analysis

**DOI:** 10.1093/ckj/sfae012

**Published:** 2024-01-24

**Authors:** Lei Dong, Yuncan Hu, Dan Yang, Liu Liu, Yueqiang Li, Shuwang Ge, Ying Yao

**Affiliations:** Division of Nephrology, Tongji Hospital, Tongji Medical College, Huazhong University of Science and Technology, Wuhan, China; Division of Nephrology, Xiangyang Central Hospital, Affiliated Hospital of Hubei University of Arts and Science, Xiangyang, China; Division of Nephrology, Tongji Hospital, Tongji Medical College, Huazhong University of Science and Technology, Wuhan, China; Division of Nephrology, Tongji Hospital, Tongji Medical College, Huazhong University of Science and Technology, Wuhan, China; Division of Nephrology, Tongji Hospital, Tongji Medical College, Huazhong University of Science and Technology, Wuhan, China; Division of Nephrology, Tongji Hospital, Tongji Medical College, Huazhong University of Science and Technology, Wuhan, China; Division of Nephrology, Tongji Hospital, Tongji Medical College, Huazhong University of Science and Technology, Wuhan, China

**Keywords:** cohort study, IgA nephropathy, meta-analysis, microangiopathy, outcome

## Abstract

**Background:**

Microangiopathy (MA) lesions are not rare in immunoglobulin A nephropathy (IgAN) and have been suggested to have a potential role in increasing risk in renal function decline. However, this suggestion has not been universally accepted. We aimed to investigate its role in our cohort and in multiple studies through a systematic meta-analysis.

**Methods:**

This cohort study included 450 IgAN patients, confirmed by renal biopsy, at Tongji Hospital, China, from January 2012 to December 2016. Clinical data were collected and analysed. We systematically searched PubMed and Web of Science for studies investigating the association between MA lesions and IgAN.

**Results:**

In our cohort, IgAN patients with MA were significantly older and had higher blood pressure, more proteinuria, worse kidney function and increased uric acid levels compared with patients without MA. When comparing pathological features with the non-MA group, the MA group exhibited more global glomerulosclerosis and interstitial fibrosis/tubular atrophy. MA lesions were independently associated with a composite kidney outcome in IgAN patients {adjusted hazard ratio 2.115 [95% confidence interval (CI) 1.035–4.320], *P* = .040}. Furthermore, this relationship was validated in a meta-analysis involving 2098 individuals from five independent cohorts. The combined data showed a 187% adjusted risk of poor renal outcome in IgAN patients with MA compared with patients without MA [adjusted risk ratio 2.87 (95% CI 2.05–4.02; *I*^2^ = 53%).

**Conclusion:**

MA lesions could serve as a valuable predictor for disease progression in patients with IgAN, extending beyond the widely recognized Oxford MEST-C score.

KEY LEARNING POINTS **What was known:**

- Immunoglobulin A nephropathy (IgAN) is the most prevalent glomerulonephritis globally.
- The MEST-C score from the Oxford classification has commonly been used to forecast the prognosis of IgAN.
- Microangiopathy is frequently observed in IgAN, but its role remains obscured.

**This study adds:**

- Microangiopathy independently correlates with kidney composite outcome alongside the MEST-C score in our cohort. Moreover, this association was further confirmed in a meta-analysis involving five cohorts from different centres.

**Potential impact:**

- Microangiopathy could serve as a valuable indicator for predicting the prognosis of IgAN.

## INTRODUCTION

Immunoglobulin A nephropathy (IgAN) is the most common glomerulonephritis worldwide, particularly prevalent in East Asian countries [1]. Clinical manifestations of IgAN encompass a range of syndromes, including asymptomatic haematuria with varying degrees of proteinuria, recurrent gross haematuria, rapidly progressive glomerulonephritis, nephrotic syndrome and acute kidney injury [2]. The morphological changes of kidney biopsies in IgAN patients vary and are associated with prognosis. The Oxford histologic classification updated in 2017 identified five variables that independently predict outcomes and offer prognostic insights [3]. These parameters form the MEST-C score, comprising mesangial hypercellularity (M), endocapillary cellularity (E), segmental sclerosis (S), interstitial fibrosis/tubular atrophy (T) and crescents (C) [3]. However, it is noted that the MEST-C score does not encompass all the common pathological changes observed in IgAN.

Renal arterial and arteriolar lesions have been strongly linked to blood pressure (BP) and glomerular filtration rate (GFR) in IgAN, although their association with proteinuria and combined kidney failure events is less pronounced [4]. These lesions were under consideration for inclusion in the Oxford classification during the preparatory phase but were ultimately not included in the final version [4, 5].

Thrombotic microangiopathy (TMA), characterized by pathological features representing tissue responses to endothelial injury, is a form of intrarenal vascular lesions that has been consistently observed in cases of IgAN [6]. Notably, not all microangiopathies exhibit thrombosis, leading a recent Kidney Disease: Improving Global Outcomes (KDIGO) conference to propose referring to the process as microangiopathy (MA), encompassing lesions with and without thrombi [7]. Although some retrospective studies have described the prevalence of MA in IgAN and evaluated its impact on disease progression, these studies have been conducted at various single centres and lack systematic multicentre evaluation [6, 8–13].

In our present study, we analysed the prevalence, clinical characteristics and role of MA in 450 IgAN patients at our centre. Additionally, we conducted a meta-analysis of studies from multiple centres to provide a comprehensive evaluation of MA in IgAN.

## MATERIALS AND METHODS

### Study design and participants

**Figure 1:**
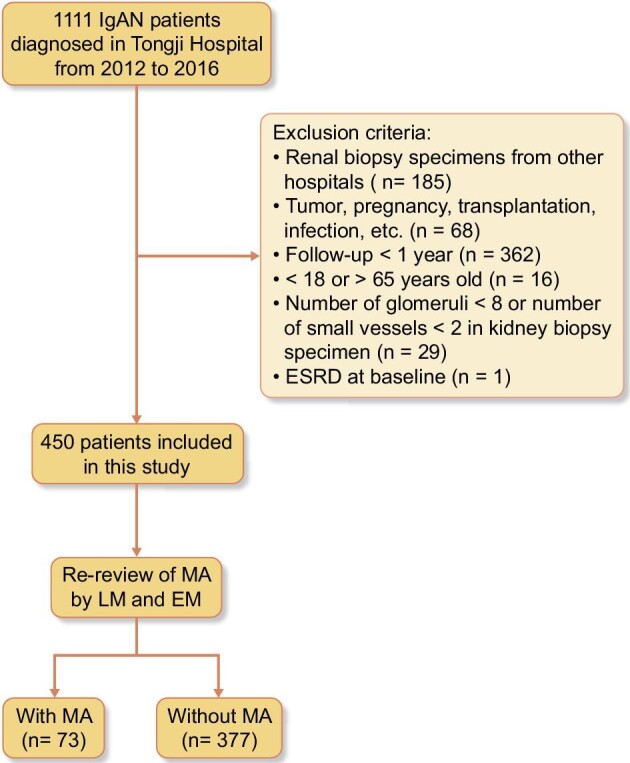
Flow chart of participant selection in this study. ESRD: end-stage renal disease.

We conducted a retrospective cohort study at Tongji Hospital in Wuhan, from 2012 to 2016, including IgAN patients confirmed by native kidney biopsy. A total of 1111 participants with IgAN were initially enrolled, but ultimately only 450 patients were included (Fig. 1). Biopsies from transplanted kidneys, inadequate biopsies, specimens from other hospitals and biopsies from patients who were pregnant or had an infection or tumour were excluded. Additionally, patients <18 years or >65 years, as well as those with <1 year of follow-up, were also excluded.

Due to the retrospective nature of the study, informed consent was waived and the protocol was approved by the Ethics Committee of Tongji Hospital, Tongji Medical College, Huazhong University of Science and Technology (TJ-IRB20211287).

### Clinical data collection

Clinical information, including demographic, anthropometric and biochemical parameters, was collected at the time of kidney biopsy. The following data were retrieved from the electronic medical records: age, gender, BP, history of hypertension, proteinuria, blood urea nitrogen (BUN), serum creatinine (SCr), estimated GFR [eGFR; according to the Chronic Kidney Disease Epidemiology Collaboration (CKD-EPI) equation], uric acid, haemoglobin, platelet count, white blood cell (WBC) count, red blood cell (RBC) count, albumin, total cholesterol, triglyceride, serum C3 and serum C4. Additionally, the use of steroids and other immunosuppressants (cyclophosphamide, mycophenolate mofetil, tacrolimus and cyclosporine A) was recorded during the follow-up period.

### Renal biopsy evaluation

All kidney biopsy specimens were processed for light microscopy (LM), immunofluorescence staining and electron microscopy (EM). Slides analysed for light microscopy were stained by haematoxylin and eosin (H&E), Masson's trichrome, periodic acid–Schiff and silver stain using standard protocols. For immunofluorescence microscopy, staining included anti-IgA, IgG, IgM, C3, C1q, κ and γ light chains and fibrinogen. The intensity of immunofluorescence was graded as negative, 1+, 2+, 3+ and 4+. The biopsies were reviewed by two experienced pathologists (L.L. and Y.L.) and classified according to the Oxford MEST-C score of IgAN [3, 4]. Parameters including M, E, S, T and C were scored based on the recommendations developed by the IgA Nephropathy Classification Working Group [3].

**Figure 2:**
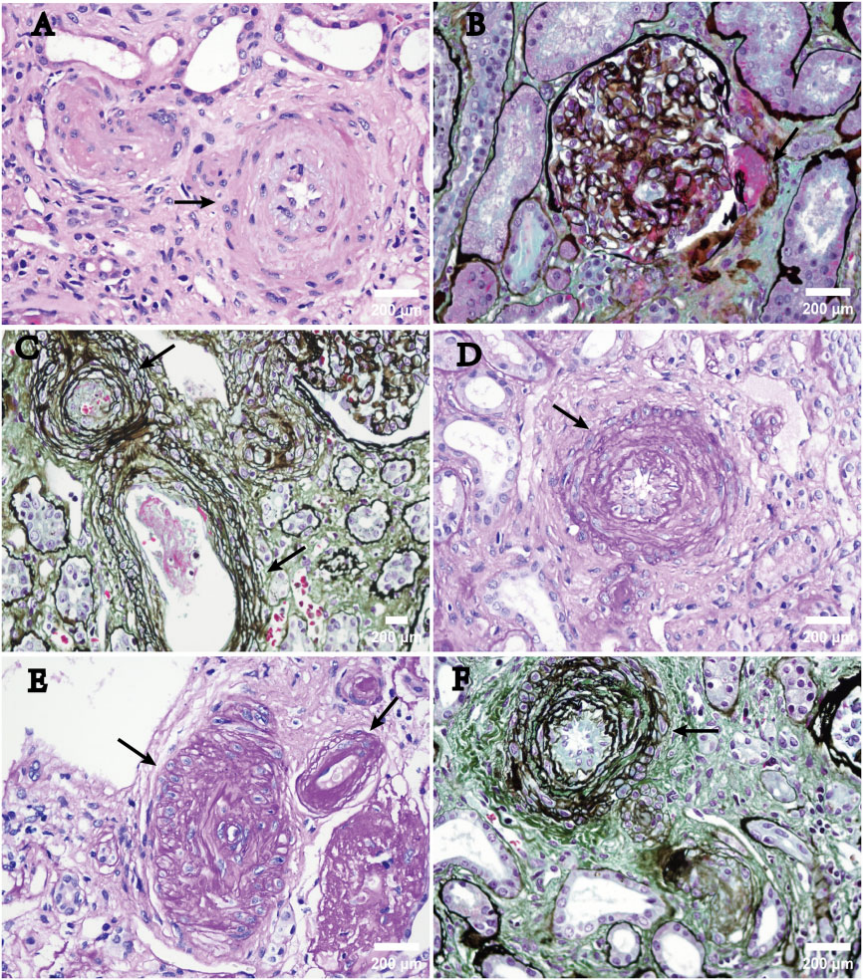
Typical images of MA in IgAN under light microscopy. (**A**) Intimal mucoid degeneration resulting in stenosis and occlusion of the vascular lumen (H&E, ×400). (**B**) Thrombosis in the afferent glomerular arteriole (periodic acid–Schiff, ×400). (**C**) Reduplication of the internal elastic lamina in an interlobular artery, showing an ‘onion skin’ appearance (periodic acid–Schiff, ×200). The upper arrow indicates endothelial cell swelling, RBC debris and artery occlusion. The arrow below points to a fibrinous thrombus. (**D**) Thickening and stratification of the inner elastic lamina, endothelial cell swelling and severe lumen stenosis (H&E, ×400). (**E**) Fibrous thickening of the intima, almost complete occlusion of the artery (left arrow) and hyaline degeneration of the arteriole (right arrow) (H&E, ×400). (**F**) ‘Onion skin’ lesions and endothelial cell swelling (periodic acid–Schiff, ×400).

The pathological features of MA were categorized into active and chronic lesions. Active lesions encompassed thrombi, endothelial swelling or denudation, mesangiolysis and fragmented red blood cells located in glomeruli, arteries or arterioles [7]. Chronic lesions included double contours of peripheral capillary walls in glomeruli, hyaline deposits in arterioles and fibrous intimal thickening with concentric lamination (onion skin) in arteries [7]. It is noted that while hyaline arteriolar deposits are commonly observed in chronic MA lesions, isolated hyaline arteriolar deposits alone could not be considered as diagnostic of MA lesions. Subendothelial flocculent material, new subendothelial basement membrane and widening of the subendothelial zone by EM were also defined as features of MA lesions [7]. Typical images without and with MA are presented in Fig. 2.

### Outcomes

The composite kidney outcome was defined as a combined event, including a decrease in eGFR ≥50%, end-stage renal disease, kidney transplantation or death. Patients who did not reach the endpoint or were lost to follow-up were censored. Survival time was calculated from the date of renal biopsy to the date of last visit, the occurrence of the endpoint or 31 December 2022.

### Literature search strategy and selection criteria

We conducted a systematic review of the published literature to carry out a meta-analysis. We identified relevant studies by searching PubMed and Web of Science using specific terms such as ‘thrombotic microangiopathy’, ‘microangiopathy’, ‘TMA’, ‘MA’, ‘IgA’, ‘IgAN’, ‘IgA nephropathy’, ‘IgA nephritis’ and ‘Berger's disease’. The search was limited to studies exploring the significance of MA lesions in IgAN until 30 November 2023. Eligible studies had to meet the following criteria: the study design was either case–control, cohort or clinical trial; the focus of interest was TMA or MA lesions; the outcome included composite kidney endpoint; and parameters including the hazard ratio (HR), odds ratio (OR) or risk ratio (RR) and the corresponding 95% confidence interval (CI) were available. The Preferred Reporting Items for Systematic Reviews and Meta-Analyses flow chart of the meta-analysis is included in Supplementary Fig. S1.

The literature search, data extraction and quality assessment were performed independently by two authors (L.D. and D.Y.). Any discrepancies between the two investigators were adjudicated by a third reviewer (S.G.).

### Statistical analysis

We checked continuous variables for normal distribution and presented them as mean ± standard deviation (SD; normally distributed variables) or as median with interquartile range (IQR; non-normally distributed variables). Categorical variables were expressed as percentages. Independent sample *t*-test and Mann-Whitney U test were applied for comparisons of normally and non-normally distributed variables, respectively.

Renal survival curves were generated by the Kaplan–Meier method and further compared by the logrank test. Univariate and multivariate Cox proportional hazards models were utilized to evaluate the association between MA and renal outcomes. Model performance was evaluated using the concordance index (C-index).

All statistical tests were conducted using SPSS software (version 24.0; IBM, Armonk, NY, USA) and R software (R Foundation for Statistical Computing, Vienna, Austria).

Meta-analysis was conducted using Review Manager (version 5.3; Cochrane, London, UK). Heterogeneity of the RR across studies was assessed with the *I*^2^ statistic test. *I*^2^ values were graded

as insignificant (0–25%), low (26–50%), moderate (51–75%) and high (>75%) heterogeneity. *P*-values <.05 indicated a statistically significant difference.

## RESULTS

### Clinical characteristics of the cohort

This study included a total of 450 IgAN patients, with a mean age of 35.4 years (SD 10.1) and men accounting for 42.2% of the participants (Table 1). All participants were of Asian descent. The cohort exhibited a median proteinuria of 0.8 g/day at baseline. The median follow-up duration was 5 years, during which 84.7% of patients received treatment involving steroids and immunosuppressants. A total of 11.1% of patients reached the composite endpoint. Among our patients, 16.2% presented acute or organized MA lesions.

**Table 1:** Characteristics of IgAN patients with or without MA.

Characteristics	All patients (*N* = 450)	MA (*n* = 73)	No MA (*n* = 377)	*P*-value
Clinical information				
Age (years)	35.4 ± 10.1	37.6 ± 10.7	35.0 ± 10.0	.041*
Male, *n* (%)	190 (42.2)	32 (43.8)	158 (41.9)	.760
SBP (mmHg)	127.7 ± 18.5	132.1 ± 19.9	126.8 ± 18.1	.026*
DBP (mmHg)	82.4 ± 13.4	83.1 ± 13.6	83.4 ± 13.4	.614
Hypertension history, *n* (%)	173 (38.4)	33 (45.2)	140 (37.1)	.195
Malignant hypertension, *n* (%)	5 (1.1)	3 (4.1)	2 (0.5)	.032*
Connective tissue disease, *n* (%)	3 (0.7)	1 (1.4)	2 (0.5)	.413
Crohn's disease, *n* (%)	0	0	0	–
Ulcerative colitis, *n* (%)	1 (0.2)	0 (0)	1 (0.3)	1.000
Hepatitis B virus infection, *n* (%)	45 (10)	7 (9.6)	38 (10.1)	.898
IgA vasculitis (Henoch–Schönlein purpura), *n* (%)	8 (1.8)	0 (0)	8 (2.1)	.365
Proteinuria (g/24 h), median (IQR)	0.8 (0.5–1.7)	1.2 (0.6–1.9)	0.8 (0.4–1.6)	.030*
SCr (μmol/l)	92.3 ± 41.0	103.9 ± 43.2	90.1 ± 40.3	.008**
eGFR (ml/min/1.73 m^2^)	88.8 ± 29.9	77.9 ± 29.4	91.0 ± 29.6	<.001**
Uric acid (μmol/l)	351.7 ± 105.4	385.0 ± 100.7	345.3 ± 105.2	.003**
WBC count (×10^9^/l)	7.17 ± 2.29	7.53 ± 2.88	7.11 ± 2.15	.243
RBC count (×10^9^/l)	4.39 ± 0.65	4.40 ± 0.65	4.39 ± 0.65	.940
Haemoglobin (g/l)	129.7 ± 19.2	129.0 ± 19.0	129.8 ± 19.2	.732
Platelet count (×10^9^/l)	229.4 ± 67.1	241.9 ± 63.1	227.0 ± 67.6	.082
Albumin (g/l)	39.3 ± 5.6	39.1 ± 4.7	39.3 ± 5.8	.824
Total cholesterol (mmol/l)	4.6 ± 1.2	4.8 ± 1.1	4.6 ± 1.3	.215
Triglyceride (mmol/l)	1.9 ± 1.4	2.0 ± 1.2	1.9 ± 1.4	.580
Serum C3 (g/l)	1.0 ± 0.4	1.0 ± 0.2	1.0 ± 0.4	.866
Serum C4 (g/l)	0.2 ± 0.1	0.2 ± 0.1	0.2 ± 0.1	.405
Duration from onset to biopsy (months), median (IQR)	6 (2–20)	6 (2–13)	6 (2–24)	.451
Follow-up time (months), median (IQR)	60 (41–76)	57 (33–71)	60 (42–76)	.134
Medications, *n* (%)				
Use of steroids and other immunosuppressants	381 (84.7)	64 (87.7)	317 (84.1)	.436
Use of RAASis	249 (55.5)	41 (56.2)	208 (55.3)	.894
Outcome				
Achieve composite outcome, *n* (%)	50 (11.1)	13 (17.8)	37 (9.8)	.047*

Values are presented as mean ± SD unless stated otherwise.

* *P* < .05; **P < .01.

When comparing clinical characteristics between IgAN patients with or without MA, the group with MA exhibited significantly older age, more serious proteinuria, higher systolic BP (SBP) and higher BUN, SCr and uric acid levels. However, no significant difference was observed in terms of gender, diastolic BP (DBP), history of hypertension, haemoglobin, platelet count, WBC count, RBC count, albumin, total cholesterol, triglyceride, serum C3, serum C4, follow-up duration and use of medications between the two groups. Furthermore, a significantly higher number of patients in the MA group reached the composite endpoint.

We conducted a subgroup analysis within the MA group based on the participants’ BP upon admission, defining uncontrolled BP as ≥140/90 mmHg. Compared with the IgAN patients with MA lesions under controlled BP, those with uncontrolled BP upon admission showed significantly worse kidney function and tended to have a poorer kidney outcome, although this trend was not statistically significant (Supplementary Table S1). Additionally, no significant differences were observed in terms of proteinuria, albumin and uric acid levels between the two groups (Supplementary Table S1).

### Histologic findings in IgAN patients with or without MA

In the evaluation of pathological features between the two groups, as presented in Table 2, it was notable that participants in the MA group showed a higher percentage of global glomerulosclerosis, interstitial fibrosis and arteriolar hyalinosis. Additionally, fewer patients in the MA group exhibited IgG deposits in the glomerulus. However, there were no significant differences between the groups regarding the presence of podocytopathies; the percentages of M1, E1, S1 and C1/C2; the intensity of IgA deposits and the percentage of arterial intimal fibrosis.

**Table 2:** Comparison of pathological features between groups.

Features	All patients (*N* = 450)	MA (*n* = 73)	Without MA (*n* = 377)	*P*-value
Total glomeruli, median (IQR)	16 (12–21)	15 (11–21)	16 (12–22)	.326
Global glomerulosclerosis (%), median (IQR)	12.5 (4.3–28.8)	22.7 (7.8–50.0)	11.8 (3.9–27.1)	<.001**
Podocytopathies, *n* (%)	45 (10.0)	11 (15.1)	34 (9.0)	.115
Oxford classification score, *n* (%)				
M1	213 (47.3)	38 (52.1)	175 (46.4)	.377
E1	258 (57.3)	37 (50.7)	221 (58.8)	.201
S1	363 (80.7)	63 (86.3)	300 (79.6)	.183
T1/T2	115 (25.6)/60 (13.3)	23 (31.5)/17 (23.3)	92 (24.4)/43 (11.4)	.004**
C1/C2	149 (33.1)/15 (3.3)	24 (32.9)/3 (4.1)	125 (33.2)/12 (3.2)	.922
IF staining, *n* (%)				
IgA				
1–2+	251 (55.8)	39 (53.4)	212 (56.2)	.658
3–4+	199 (44.2)	34 (46.6)	165 (43.8)	
IgG	96 (21.3)	22 (30.1)	74 (19.6)	.045*
IgM	179 (39.8)	31 (42.5)	148 (39.3)	.608
C3c	392 (87.1)	63 (86.3)	329 (87.3)	.822
C4c	7 (1.6)	3 (4.1)	4 (1.1)	.054
Arterio-/arteriolosclerosis, *n* (%)				
Arterial intimal fibrosis	178 (39.6)	34 (46.6%)	144 (38.2%)	.180
Arteriolar hyalinosis	109 (24.2)	28 (38.4%)	81 (21.5%)	.002**

* *P* < .05, **P < .01.

### MA independently correlates with poor outcome

**Figure 3:**
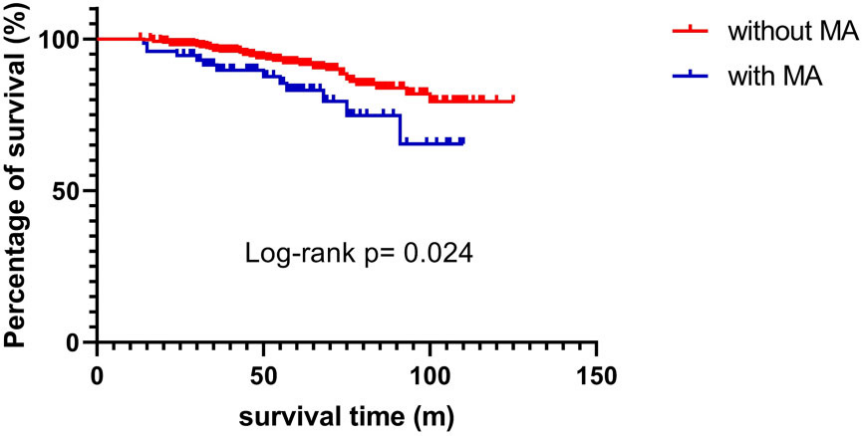
Kaplan–Meier curve for the composite outcome according to the presence of MA lesions.

Table 3 provides a univariate analysis of the various clinical and pathological parameters related to poor outcomes in IgAN patients. Among all the parameters, SBP, DBP, mean arterial pressure (MAP), SCr, uric acid, proteinuria, RBC count, albumin, M1 and T1/T2 were found to correlate with the composite endpoint. As anticipated, MA was significantly associated with poor outcome. A better survival rate was achieved in patients without MA rather than patients with MA (logrank test, *P* = .024; Fig. 3) when applied in the Kaplan–Meier analysis.

**Table 3:** Predictors for the composite outcome by univariate Cox regression analysis.

Variants	HR	95% CI	*P*-value
Clinical features			
Gender	0.749	0.430–1.305	.308
Age	1.004	0.976–1.032	.801
SBP	1.025	1.010–1.039	.001**
DBP	1.026	1.006–1.046	.011*
MAP	1.030	1.011–1.049	.002**
SCr	1.015	1.012–1.018	<.001**
Uric acid	1.005	1.003–1.007	<.001**
Proteinuria	1.167	1.086–1.254	<.001**
WBC	0.995	0.882–1.122	.933
RBC	0.336	0.215–0.525	<.001**
Platelet	0.998	0.994–1.002	.404
Albumin	0.916	0.884–0.949	<.001**
TC	1.085	0.905–1.300	.379
Use of steroids and other immunosuppressants	1.159	0.493–2.726	.735
Pathological features			
M1	1.814	1.018–3.233	.043*
E1	1.319	0. 740–2.350	348
S1	2.002	0.794–5.045	.141
T			<.001**
T1	3.666	1.663–8.081	.001**
T2	15.037	7.164–31.562	<.001**
C			.133
C1	1.199	0.665–2.161	.334
C2	3.200	0.963–10.631	.692
MA	2.035	1.081–3.830	.028*

*P < .05, **P < 0.01.

The contribution of MA to the poor outcome was further analysed by Cox proportional hazards modelling (Table 4). After adjusting for potential confounding factors such as sex, age, MAP, SCr, uric acid, proteinuria, RBC count, albumin, MEST-C score and use of steroids and other immunosuppressants, MA was found to independently correlate with poor outcome (Table 4). It is noteworthy that the models demonstrated good performance, with a C-index >0.8 (Table 4 and Supplementary Table S2). However, when examining MA lesions categorized as acute lesions only, chronic lesions only and coexistence of acute and chronic lesions, no significant associations between these lesion categories and renal outcomes were observed (Supplementary Table S3), which may be attributed to the limited size of our sample.

**Table 4:** MA independently predicts bad outcome in IgAN patients.

MA	HR (95% CI)	*P*-value	C-index (95% CI)
Model 1	2.068 (1.060–4.035)	0.033*	0.844 (0.773, 0.914)
Model 2	2.106 (1.031–4.302)	0.041*	0.849 (0.776, 0.921)
Model 3	2.115 (1.035–4.320)	0.040*	0.849 (0.777, 0.921)

* *P* < .05.

Model 1: adjusted for sex, age, MAP, SCr, uric acid, proteinuria, RBC count and albumin;

model 2: adjusted for model 1 plus Oxford classification (MEST-C score);

model 3: adjusted for model 2 plus use of steroids and other immunosuppressants.

### Meta-analysis

The literature search yielded 306 articles, from which four eligible studies were included (Supplementary Fig. S1). In combination with the present cohort, a total of five cohorts comprising 2098 participants were analysed in the meta-analysis. The characteristics of these five cohorts are summarized in Table 5. All of them are retrospective cohort studies conducted at different centres, with the prevalence of MA in IgAN ranging from 8.3 to 53% and a mean value of 18.8%.

**Table 5:** Characteristics of included studies.

Study	El Karoui *et al*. [9]	Cai *et al.* [6]	Neves *et al.* [12]	Ruan *et al.* [13]	Present study
Year	2012	2019	2020	2023	2023
Location	France	China	Brazil	China	China
Study design	Retrospective cohort	Retrospective cohort	Retrospective cohort	Retrospective cohort	Retrospective cohort
MA prevalence, *n*/*N* (%)	68/128 (53)	194/944 (20.6)	21/118 (17.8)	38/458 (8.3)	73/450 (16.2)
Mean age (years)	38.7	36	33	33.2	35.4
Male (%)	69.5	51	55	46.1	42.2
Mean length of follow-up (months)	44	100	65	54	60
Statistical method	Cox regression	Cox regression	Logistic regression	Cox regression	Cox regression

**Figure 4:**
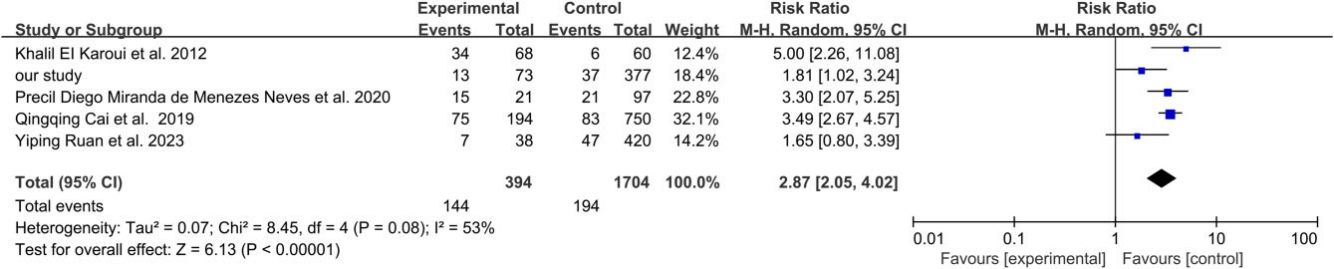
Forest plot of the included studies comparing the risk of the kidney composite endpoint between IgAN patients with and without MA lesions.

The meta-analysis combined data from five studies, with weights ranging from 12.4 to 32.1% (Fig. 4). Overall, the analysis indicated that MA lesions were independently associated with an increased risk of poor kidney outcome in IgAN patients [RR 2.87 (95% CI 2.05–4.02), *P* < .001] (Fig. 4). Moderate heterogeneity was observed (*I*^2^ = 53%, *P* = .08) (Fig. 4).

## DISCUSSION

In this study we reported that IgAN patients with MA exhibited higher levels of proteinuria, worse kidney function and elevated uric acid levels in their clinical profiles. In terms of pathological features, the MA group displayed a higher prevalence of global glomerulosclerosis and a greater proportion of interstitial fibrosis/tubular atrophy, which suggested that MA lesions are late events in IgA evolution. Furthermore, multivariate analysis revealed that MA independently correlated with the composite renal endpoint. Additionally, our comprehensive meta-analysis demonstrated a direct association between MA and a 187% adjusted risk of poor renal outcomes.

The prevalence of MA in IgAN has been shown to vary widely, ranging from 2 to 53% in previous studies [6, 8–10]. Notably, El Karoui *et al*. [9] reported the highest prevalence, potentially due to the patient population being from an active hypertension clinic, with their MA group exhibiting a markedly higher mean initial SBP (161 mmHg) compared with other cohorts. Furthermore, their study indicated that 26% of the MA group had malignant hypertension, while Cai *et al*. [6] reported an incidence of 9.8% and Chua *et al*. [11] reported 8%. It is evident that the incidence of MA in IgAN is higher in cohorts with severe hypertension. However, it is unlikely that severe hypertension is the sole cause of MA in IgAN, as numerous cases have been observed in patients with normal BP. In our cohort, the subgroup analysis conducted in the MA group suggested a potential role of uncontrolled BP in exacerbating kidney function, but not in relation to proteinuria. Nonetheless, further investigation in larger cohorts is warranted to validate these findings.

It is well-documented that hyperuricaemia is common in IgAN and has been identified to be an independent risk factor of disease progression [14, 15]. In the body, two-thirds of the uric acid is expelled by the kidneys and the remainder is eliminated by the gastrointestinal tract [16]. All uric acid is initially filtered by the glomerulus, with ≈99% being reabsorbed in the proximal tubule. Subsequently, excretion and post-secretory reabsorption take place at different segments of the proximal tubule in subsequence [16]. Hyperuricaemia had a strong correlation with the severity of tubulointerstitial lesions [17]. In the current study, our findings revealed that the MA group exhibited significantly higher levels of uric acid and more severe interstitial fibrosis/tubular atrophy compared with the non-MA group. This could potentially be explained by the impaired capacity of expelling uric acid due to chronic tubulointerstitial lesions in the MA group.

Interestingly, there appears to be less laboratory evidence of MA in the patients with MA morphologic changes. In El Karoui et al.’s study [9], only 12.3% of the MA group presented with laboratory evidence of MA. They reported that MA with laboratory evidence was independently correlated with a decline of eGFR and poor outcomes, whereas the presence of only morphologic MA did not show the correlation. However, it is worth noting that IgAN patients with only morphologic MA had significantly shorter survival times compared with IgAN patients without MA [6, 12]. Our findings also indicate that histologic MA is significantly correlated with poor renal function outcomes. Moreover, the results of a meta-analysis further reinforce the correlations between histologic MA and kidney composite endpoint.

The Oxford classification has been widely accepted and validated across various populations as a valuable tool. However, it should be noted that it does not serve as a substitute for a full, detailed pathology report. Our cohort revealed that the presence of MA lesions was a significant predictor of kidney composite endpoint, independent of the MEST-C score. Our meta-analysis, which incorporated studies from different countries, further demonstrated a strong association between MA lesions and disease progression. Based on these compelling findings, we propose the inclusion of MA lesions in the Oxford classification to enhance the prognostic value for IgAN.

It is unknown whether IgAN with MA is a coincidence of two diseases or has an intrinsic relationship. However, there are indications that endothelial cell injury could be a common underlying mechanism of TMA, which consists of a group of different disorders, leading to microvasculature thrombosis [18]. Dysregulated complement activation has been implicated in TMA disorders such as haemolytic uraemic syndrome and thrombotic thrombocytopenic purpura, leading to endothelial cell injury [19]. Mutations in complement factors associated with IgAN have been reported in several studies [20–22]. Trials of ravulizumab, a complement inhibitor, are under way for patients with IgAN [23]. In addition, variants of complement factor H have been described to be associated with MA lesions in IgAN [24]. Interestingly, in our cohort study, we did not find a difference in glomerular deposits of C3 between patients with and without MA, which is inconsistent with other research [12]. However, C4d deposits have been associated with renal function decline in IgAN with MA [11, 25]. The overwhelming presence of C4d deposits without co-deposition of C1q in IgAN patients with MA suggests a potential role of the lectin pathway in the development of MA in IgAN [12, 18].

This study has several limitations that should be acknowledged. First, the retrospective and observational design limited our ability to collect comprehensive data, such as haemolytic markers and schistocytes, which would have allowed us to investigate clinical manifestations of MA in this cohort. Another limitation is the relatively low use of renin–angiotensin–aldosterone inhibitors (RAASis) in our cohort (55.5%) compared with other retrospective studies, which varied from 40 to 97.4% [6, 11, 12]. This discrepancy in medication use may have influenced the prognosis of IgAN patients in our cohort, highlighting the need for prospective studies to assess the impact of RAASis on disease outcomes. Furthermore, the findings of this study are based on a single Chinese centre, which may limit their generalizability to other populations. To address this limitation, we conducted a meta-analysis that included cohorts from different countries, increasing the robustness and power of our findings.

In our cohort we observed MA lesions in 16.2% of IgAN patients, and this subgroup demonstrated higher BP, increased proteinuria and poorer kidney function when compared with those without MA lesions. Notably, MA lesions were found to be an independent predictor of an unfavourable renal outcome in our cohort, and this association was further confirmed through a meta-analysis comprising various multicentre cohorts. These findings significantly underscore the substantial role of MA lesions as an independent prognostic predictor for IgAN.

## Supplementary Material

sfae012_Supplemental_File

## Data Availability

The data underlying this article will be shared upon reasonable request to the corresponding author.
