# Supplementary material for: Microangiopathy associated with poor outcome of immunoglobulin A nephropathy: a cohort study and meta-analysis
Source: Clin Kidney J. 2024 Jan 24;17(2):sfae012. doi: 10.1093/ckj/sfae012 (PMC10851670; doi:10.1093/ckj/sfae012)
Supplement: sfae012_Supplemental_File [file sfae012_supplemental_file.docx]

Records identified (n= 306):

PubMed (n= 105), Web of Science (n= 201)

Studies included in the meta-analysis (n= 5)

The present study (n= 1)

Eligible studies (n= 4)

Excluded by full text (n= 21):

did not evaluate associations between TMA with renal outcome

Full text analysis (n= 25)

Excluded by screening titles and abstracts (n= 201):

not original study (eg. review, letter), basic research study

Duplicate records removed (n= 80)

Records screened (n= 226)

Supplement figure 1. Flow chart of illustrating the study selection process. Abbreviations: TMA, thrombotic microangiopathy.

Supplement table 1. Comparison of clinical features between IgAN patients with TA lesions based on their BP at admission.

|  | Uncontrolled BP on admission  (n= 26) | Controlled BP on admission  (n= 47) | P value |
| --- | --- | --- | --- |
| Clinical Information |  |  |  |
| Age, years | 41.2 ± 11.3 | 35.6 ± 9.8 | 0.040^*^ |
| Male, n (%) | 18 (69.2) | 14 (29.8) | 0.001^**^ |
| Systolic blood pressure (mmHg) | 151.4 ± 17.8 | 121.4 ± 10.9 | < 0.001^**^ |
| Diastolic blood pressure (mmHg) | 92.3 ± 15.1 | 78.1 ± 9.5 | < 0.001^**^ |
| Proteinuria, g/24 h | 1.5 (0.5- 2.4) | 1.0 (0.5- 1.7) | 0.228 |
| Scr, μmol/L | 121.6 ± 54.3 | 94.1 ± 32.3 | 0.032^*^ |
| Uric acid, μmol/L | 409.8 ± 96.4 | 371.3 ± 101.3 | 0.158 |
| Albumin, g/L | 39.4 ± 6.1 | 39.0 ± 3.9 | 0.633 |
| Outcome |  |  |  |
| Achieve composite outcome, n (%) | 8 (30.8) | 5 (10.6) | 0.067 |

^*^ *P* < 0.05; ^**^ *P* < 0.01

Supplement table 2. Validation of models using the C-index among Cox regression models with and without MA-lesions.

|  | C-index (95% CI) |  | C-index (95% CI) | *P* value |
| --- | --- | --- | --- | --- |
| Model 1 + MA | 0.844 (0.773, 0.914) | Model 1 | 0.841 (0.772, 0.909) | 0.039^*^ |
| Model 2 + MA | 0.849 (0.776, 0.921) | Model 2 | 0.848 (0.777, 0.918) | 0.043^*^ |
| Model 3 + MA | 0.849 (0.777, 0.921) | Model 3 | 0.847 (0.776, 0.917) | 0.038^*^ |

C-index concordance index; CI, confidence interval.

^*^ *P* < 0.05; ^**^ *P* < 0.01

Model 1 + MA: Cox regression model adjusted for MA-lesions, sex, age, MAP, Scr, uric acid, proteinuria, RBC and albumin.

Model 2 + MA: Cox regression model adjusted for model 1+ MA plus Oxford classification (MEST-C score).

Model 3+ MA: Cox regression model adjusted for model 2+ MA plus use of steroids and other immunosuppressants.

Model 1: Cox regression model adjusted for sex, age, MAP, Scr, uric acid, proteinuria, RBC and albumin.

Model 2: Cox regression model adjusted for model 1 plus Oxford classification (MEST-C score).

Model 3: Cox regression model adjusted for model 2 plus use of steroids and other immunosuppressants.

Supplement table 3. Summary of Cox regression models for composite endpoint in IgAN with different stages of MA lesions.

|  | Univariate |  | Model 1 |  | Model 2 |  | Model 3 |  |
| --- | --- | --- | --- | --- | --- | --- | --- | --- |
|  | HR (95% CI) | *P* value | HR (95% CI) | *P* value | HR (95% CI) | *P* value | HR (95% CI) | *P* value |
| Acute MA lesions only (n= 30) | 1.349（0.430，4.235） | 0.608 | 2.969（0.311，28.370） | 0.345 | 0.298（0.007，12.003） | 0.521 | 0.697（0.013，38.381） | 0.860 |
| Chronic MA lesions only (n= 37) | 1.174(0.380,3.624) | 0.781 | 2.133（0.383，11.884） | 0.387 | 3.983（0.130，122.217） | 0.429 | 2.150（0.052，88.483） | 0.686 |
| Coexistence of both lesions (n= 6) | 0.041（0.000，109.548） | 0.427 | / | 0.988 | / | 0.931 | / | 0.951 |

HR, hazards ratio; CI, confidence interval; MA, microangiopathy.

^*^ *P* < 0.05; ^**^ *P* < 0.01

Model 1: adjusted for sex, age, MAP, Scr, uric acid, proteinuria, RBC and albumin.

Model 2: adjusted for model 1 plus Oxford classification (MEST-C score).

Model 3: adjusted for model 2 plus use of steroids and other immunosuppressants.
